# Factors associated with the utilization of community-based diabetes management care: A cross-sectional study in Shandong Province, China

**DOI:** 10.1186/s12913-020-05292-5

**Published:** 2020-05-11

**Authors:** Jingjing Yao, Haipeng Wang, Jia Yin, Di Shao, Xiaolei Guo, Qiang Sun, Xiao Yin

**Affiliations:** 1 School of Health Care Management, NHC Key Laboratory of Health Economics and Policy Research, Shandong University, Wenhuaxi Road, Lixia District, Jinan, 250012 China; 2 Shandong Centers for Disease Control and Prevention, Jingshi Road, Lixia District, Jinan, 250012 China; 3 Shandong University Affliated Jinan Center Hospital, Jiefang Road, Lixia District, Jinan, 250012 China

**Keywords:** Type-2 diabetes mellitus, Diabetes management, Healthcare utilization, Essential public health, China

## Abstract

**Background:**

Community-based diabetes management is known to be an important strategy for global diabetes control. In China, community-based diabetes management care, including regular blood glucose tests and guidance on medicine use, dietary control, and physical exercise provided by primary health institutions (PHIs), as one of the key contents of the national essential public health services (EPHS), was implemented since 2009 when the new round of health system reform was initiated. This study aimed to investigate the utilization of community-based diabetes management care services, and explore the factors influencing utilization from both patients’ and providers’ points of view.

**Methods:**

In total, 2520 type-2 diabetes mellitus (DM) patients registered for EPHS were selected from 63 PHIs in eight counties of Shandong province, China, using multi-stage stratified sampling. Of those, 2166 patients (response rate: 85.4%) completed face-to-face structured questionnaires on their utilization of community-based diabetes management care services. Further, 63 PHIs were surveyed on diabetes care delivery, and 444 primary healthcare providers were purposively sampled from those PHIs to measure their knowledge of diabetes management care delivery, using a self-developed questionnaire. Descriptive statistics were used to analyze the delivery and utilization of diabetes management care services. Multilevel logistic regression models were used to analyze the factors associated with patients’ utilization of diabetes management services.

**Results:**

All 63 PHIs reported that all the required four diabetes management services were provided through EPHS. However, only 49.6% of the patients reported they fully used these services, with no statistically significant difference between urban and rural patients. Patients who had higher knowledge of diabetes and better self-efficacy in controlling the condition, were more likely to fully utilize diabetes management care. A larger number of PHI health staff per 1000 population was associated with better utilization of care.

**Conclusion:**

Although community-based diabetes management services are well available to Chinese DM patients under the framework of EPHS, the actual utilization of diabetes management services among the patients was poor. The size of the PHI workforce, patients’ knowledge and self-efficacy in controlling diabetes, were important predictors of utilization, and could be enhanced to improve control of diabetes.

## Background

Diabetes mellitus (DM) has become one of the most common non-communicable diseases globally and is one of the most challenging public health issues [1]. The International Diabetes Federation has estimated that 425 million people worldwide had diabetes in 2017, and this is expected to rise to 627 million by 2045 [2]. China has the highest number of DM patients in the world, accounting for 25% of DM patients globally in 2013. Prevalence of DM increased from 0.9% in 1981 to 9.7% in 2008, and 11.6% in 2013, the latest available data at the time of the study [3–5]. DM is associated with increased risk of long-term cardiovascular disease, among other complications, and represents a fast-growing economic burden with considerable consequences for individuals, communities, and health systems [6].

Globally, community-based diabetes management, although varying in approach under different health systems [7–10], is an important strategy for the control of diabetes. A large number of programs have demonstrated the enormous role of community-based diabetes management in delaying complications and avoiding hospital admissions [11, 12]. Community-based diabetes management has great potential for improving patients’ quality of life and reducing the burden of disease in a cost-effective way [13, 14]. According to World Health Organization (WHO) recommendations [1], core components of community-based diabetes management include interventions to promote and support healthy lifestyles, medication for blood glucose control, regular exams for early detection of complications and standard criteria for referral of patients from primary to secondary care.

In April 2009, China initiated its new round of health system reform. The equalization of essential public health services (EPHS), as one of five key reform components, is realized through 11 community-based health services, including the provision of healthcare services for diabetes patients. To qualify for care, patients should be aged 35 years or older, and have a diagnosis of type-2 DM. These broad criteria allow for the enrolment of as many patients as possible. According to national guidelines for the implementation of EPHS, community-based diabetes management services mainly include regular blood glucose tests and guidance on medicine use, daily dietary control, and physical exercise. Primary health institutions (PHIs), which include village clinics (VCs) and township health centers (THCs) in rural counties, and community health centers and stations (CHCs and CHSs) in urban areas, should provide these services free of charge to all included DM patients at least once a quarter by appointments or home visits.

Despite the numerous benefits of community-based diabetes management on the control of diabetes, and the increasing availability of guidelines and standards for diabetes management globally, studies have consistently found a big difference between recommended services and those patients actually utilized in nearly all countries, especially in low- and middle-income countries [15–17]. In China, community-based diabetes management has been provided under the EPHS framework for the past 10 years. The delivery of those services to communities, and the extent to which diabetes patients utilize those services need to be systematically studied. Moreover, the factors contributing to the gap between delivery and utilization of community-based diabetes management care should be identified to improve its effects, ultimately strengthening evidence-based diabetes control across the globe.

Earlier studies focused on the utilization of diabetes management services among DM patients in rural China [18–20], and their results indicated that rural patients’ access to diabetes management care had been greatly improved by the implementation of EPHS. However, the discrepancy in utilization between urban and rural areas has not been thoroughly studied. Furthermore, most previous studies focused on the analysis of individual and household factors, with limited inclusion of provider factors [21, 22]. Some studies analyzed the association between patients’ utilization of diabetes management care and providers’ capacity, strength of the health workforce and EPHS financing, using in-depth interviews and theoretical analysis [23, 24]. The quantitative relationship between provider-related factors and patients’ utilization of diabetes management care has not been examined. Based on prior research, the current study aimed to study the utilization of community-based diabetes management care services in both urban and rural China, and explore the possible factors influencing utilization, drawing on the perceptions of both patients and providers.

## Methods

### Study design and setting

This was a cross-sectional study conducted in Shandong province in eastern China. The province contains 17 prefectures and 140 counties or districts, with a population of nearly 99 million (7.2% of mainland China) in 2016, comprising an urban population of 49 million and a rural population of 50 million people, ranking third on population size in the country. In 2016, Shandong’s gross domestic product (GDP) per capita was 67,706 Yuan (around 10,105 US$). There were an estimated 980,000 type-2 DM patients in the province (prevalence: 9.3%) in 2013 [25]. Further, nearly 30% of type-2 DM patients in Shandong had been enrolled in EPHS in 2016 [26]. Shandong epitomizes China in terms of population and level of economic development.

### Sampling

**Fig. 1:**
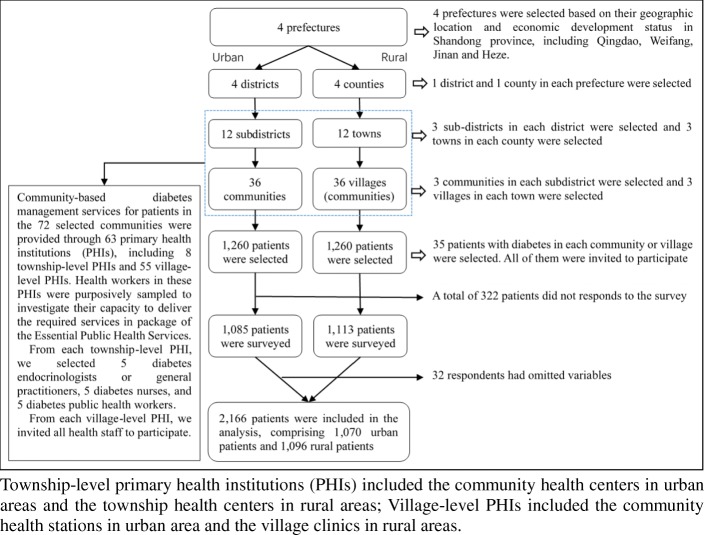
Flowchart of our sampling method

We employed multi-stage, stratified, randomized sampling to select patients registered in the EPHS Non-communicable Disease Management System (NCDMS) in Shandong. A flowchart of the sampling process is provided in Fig. 1*.* First, four representative prefectures were selected based on their geographic location (east, central and west) and economic development status within the province. Three urban subdistricts and three rural towns were randomly selected from the four prefectures. Three communities from each subdistrict and three villages from each town were randomly selected. Finally, 35 type-2 DM patients were randomly recruited from each selected community and village, with a total of 2520 patients selected for participation. The inclusion criteria were registration on NCDMS, diagnosis of type-2 DM based on WHO criteria for more than 6 months [27], aged under 80 years, and ability to communicate and understand instructions.

Community-based diabetes management services for patients in the 72 selected communities were provided through 63 PHIs, including 8 township-level PHIs and 55 village-level PHIs. Health workers in these PHIs were purposively sampled to investigate their capacity to deliver the required services, as per EPHS guidelines. In general, the health staff in township-level PHIs comprised doctors, nurses, public health workers and a small number of pharmacists and medical technicians, with the total number of medical workers in each facility ranging from 50 to 100. The health staff in village-level PHIs consisted of a general practitioner (GP) or village doctor, and a small number of medical assistants, with the total number of medical workers in each facility ranging from one to five. We used different purposive sampling methods to select the participants from these two types of PHIs, mindful of the difference in the number of health workers between them. We selected five diabetes endocrinologists or GPs, five diabetes nurses and five diabetes public health workers from each township-level PHI. In village-level PHI, all health staff were invited to participate this study. For inclusion, health staff had to be working in PHIs, contracted to provide diabetes management care, and had to have more than 6 months’ experience in providing diabetes management care.

### Data collection

Data collection was conducted from August to October 2017. All selected patients were invited to complete a structured face-to-face questionnaire. The questionnaire mainly asked about the patient’s basic demographics (residence, gender, age and household income per capita), health status (duration of diagnosis), knowledge of diabetes, self-efficacy in their control of diabetes, and their utilization of community-based diabetes management care in EPHS. Patients’ knowledge on diabetes was measured using a self-designed questionnaire comprising 16 items with reference to Chinese guidelines for type-2 diabetes [28]. The full questionnaire is available in Supplementary Materials 1. The questionnaire was pilot-tested with Cronbach’s α = 0.76. Patients’ self-efficacy in their control of diabetes was measured by the Diabetes Empowerment Scale-Short Form (DES-SF) [29], which comprises eight items. Cronbach’s α of DES-SF was 0.85 in Chinese type-2 DM patients. In total, 2166 patients completed the questionnaire without omitted variables.

Sixty-three PHIs were surveyed using self-administered questionnaires, completed by institutional heads, on diabetes care delivery, including the delivery of EPHS-mandated diabetes management services – regular blood glucose tests, along with guidance on medicine use, dietary control, and physical exercise – number of medical staff members and number of type-2 DM patients registered for diabetes management. Selected heath staff at these PHIs were invited to complete a structured self-administered questionnaire assessing their knowledge of diabetes management care delivery. The questionnaire for the assessment of diabetes management knowledge was self-designed and comprised 22 items with reference to Chinese guidelines for type-2 diabetes [28]. The full questionnaire is provided in Supplementary Materials 2. The questionnaire was pilot-tested with Cronbach’s α = 0.74. A total of 444 medical workers in PHIs completed the questionnaire with full variables.

All data collectors were rigorously trained and appropriately qualified for questionnaire delivery, and quality supervisors reviewed all completed questionnaires after each interview.

### Measurements and variables

Patients’ utilization of community-based diabetes management care was assessed using self-reports on EPHS services received, in terms of both contents and quantity. Four self-report questions were for this purpose: “How often have you utilized nutrition guidance from local PHIs in the past year?”; “How often have you utilized physical exercise guidance from local PHIs in the past year?”; “How often have you utilized medication guidance from local PHIs in the past year?”; and, “How often has your blood glucose been tested by local PHIs in the past year?” Based on EPHS criteria for diabetes management care, patients’ utilization of community-based diabetes management services was classified as fully or partially utilized. Patients who reported having utilized all recommended services at least once a quarter were considered to have fully utilized the services, while the remainder were considered to have partially utilized the services.

Individual-level variables included residence (urban or rural), gender (male or female), age (< 65 or ≥ 65 years), household income level (< 2800, 2800~, 6000 ~ and ≥ 12,000 Yuan; classified by the quartile of the household income per capita), and duration of diagnosis (< 5, 6–10, > 10 years). These variables were processed as control variables in the analysis. Individual-level variables also included patients’ knowledge of diabetes and self-efficacy in control of diabetes. Patients received one point for each correct answer to the items in the diabetic knowledge questionnaire, with the total score ranging from 0 to 16, and a higher score indicating a higher knowledge level. Response to each item of DES-SF was rated on a five-point Likert scale (1 = totally disagree, 5 = totally agree). Self-efficacy scores ranged from 8 to 40, with a higher score indicating higher self-efficacy.

Three provider variables at community level were also included in the analysis: types of PHIs providing diabetes management care, classified as township-level and village-level PHIs, with former including CHCs in the urban areas and THCs in the rural areas and the later including CHSs in urban areas and VCs in rural area; the ratio of PHI medical staff to the total population served by each institution in 2016, gauging the strength of health workforce at community level; and providers’ knowledge of diabetes management services delivery, revealing their capacity to provide appropriate care. Health workers received one point for each correct answer in the diabetic knowledge questionnaire; the total score ranged from 0 to 22, with a higher score indicating a higher knowledge level. The mean scores were used to measure providers’ knowledge on diabetes management at community level.

### Data analysis

Descriptive statistics were used to describe the characteristics of the participants and the delivery and utilization of community-based diabetes management services. Chi-squared tests were performed to determine the differences in individual- and community-level variables between urban and rural areas. Two-level logistic regression models were conducted to analyze the effects of individual- and community-level variables on patients’ utilization of community-based diabetes management care in EPHS, adjusting for all variables as fixed effects and allowing for heterogeneity between communities. A series of five models was performed with Model 1 as a null model containing no explanatory variables. Intra-class correlation coefficient (ICC) was computed to examine the necessity of fitting two-level models. Model 2 included all the control variables at individual level. Model 3 and Model 4 added individual- and community-level variables, respectively, into Model 2. Model 5 added both individual- and community-level variables into Model 2. Comparing Models 3 through 5, the impacts of individual- and community-level variables on patients’ utilization of diabetes management care after controlling for each other were assessed. All data analyses were conducted using STATA version 15.0.

## Results

### The delivery of community-based diabetes management care

Among the 63 PHIs delivering community-based diabetes management services for DM patients registered for EPHS, the majority (87.3%) were village-level PHIs, with no significant difference in the types of PHIs between urban and rural areas (85.7 vs 88.6%, *P* = 0.17). The mean number of health workers per 1000 population served by PHIs was 1.46 (SD: 0.92), with no significant difference in the health workforce between urban and rural institutions (1.40 vs 1.51, *P* = 0.96). Providers’ mean diabetes knowledge score was 15.1 (SD: 1.1). There was no significant difference in diabetes knowledge between urban and rural health workers (15.3 vs 14.9, *P* = 0.34). All participating PHIs, regardless of location, had reported providing all the diabetes management services required by EPHS for registered DM patients (Table 1).

**Table 1:** The delivery of the community-based diabetes management services in Shandong, China

Characteristic	Total	Urban	Rural	*P*-value
Types of PHIs providing the community-based diabetes management services, n (%), Column				
Township-level PHIs	8 (12.7)	4 (14.3)	4 (14.3)	0.17
Village-level PHIs	55 (87.3)	24 (85.7)	31 (88.6)	
Number of health workers per 1000 population serviced by PHIs, mean ± SD	1.46 ± 0.92	1.40 ± 0.80	1.51 ± 1.03	0.96
Healthcare providers knowledge score on diabetes management delivery in PHIs, mean ± SD	15.1 ± 1.1	15.3 ± 1.3	14.9 ± 1.0	0.34
The community-based diabetes management services provided by PHIs, n (%), Row				
Dietary control instruction	63 (100)	28 (100)	35 (100)	–
Physical excise instruction	63 (100)	28 (100)	35 (100)	–
Medicine use guide	63 (100)	28 (100)	35 (100)	–
Blood glucose test	63 (100)	28 (100)	35 (100)	–

PHIs, primary health institutions; Township-level PHIs included the community health centers in the urban areas and the township health centers in rural areas; Village-level PHIs included the community health stations in urban areas and the village clinics in rural areas; SD, standard deviation

### Baseline characteristics of the patients

Among 2166 participants, the majority were female (65.4%). Mean age was 63.4 years, with 49.9% of the participants older than 65 years. The median annual household income per capita was 6000 Yuan (around 895.5 US$). Patients in urban communities had higher household income levels than those in rural areas (*P* < 0.01). With respect to duration of diabetes, 38.5% patients had been diagnosed within 5 years, 31.4% had a duration of 5 ~ 10 years, and 30.1% had a duration of more than 10 years. Urban patients had a longer duration than rural patients (*P* < 0.01).

On the whole, the mean score of diabetes knowledge was 10.6 (SD: 3.3). Urban patients scored higher than rural patients (10.8 vs 9.9, *P* < 0.01). The mean score of self-efficacy in controlling diabetes was 32.0 (SD: 5.0). No statistically significant difference in self-efficacy was observed between urban and rural patients (32.1 vs 31.8, *P* = 0.49) (Table 2).

**Table 2:** The baseline characteristics of the patients

Variables	Total N (%)	Urban n_1_ (%)	Rural n_2_ (%)	*P-* value
Observation	2166	1070	1096	
Gender				0.28
Male	749 (34.6)	382 (35.7)	367 (33.5)	
Female	1417 (65.4)	688 (64.3)	729 (66.5)	
Age groups, years				0.89
< 65	1086 (50.1)	531 (49.6)	555 (50.6)	
≥65	1080 (49.9)	539 (50.4)	541 (49.4)	
Household income per capita, Yuan				0.00^*^
< 2800	542 (25.0)	160 (15.0)	382 (34.9)	
2800~	541 (25.0)	232 (21.7)	309 (28.2)	
6000~	541 (25.0)	327 (30.6)	214 (19.5)	
≥ 12,000	542 (25.0)	351 (32.8)	191 (17.4)	
Duration of diabetes, years				0.00^*^
< 5	833 (38.5)	368 (34.4)	465 (42.4)	
5 ~ 10	680 (31.4)	344 (32.2)	336 (30.7)	
> 10	653 (30.1)	358 (33.5)	295 (26.9)	
Diabetic knowledge score, Mean ± SD	10.6 ± 3.3	10.8 ± 3.2	9.9 ± 3.5	0.00^*^
Diabetic self-efficacy score, Mean ± SD	32.0 ± 5.0	32.1 ± 5.1	31.8 ± 5.2	0.49

*Significant at *p* < 0.05; *SD* standard deviation

### Patients’ utilization of community-based diabetes management care

Among the 2166 participants, only 49.6% patients reported having fully utilizing all required diabetes management services delivered by PHIs. There was no significant difference in utilization between urban and rural patients (48.6% vs 50.6%, *p* = 0.36). For each service, 85.6% patients reported having fully utilized blood glucose tests, 69.7% dietary control instruction, 66.0% physical exercise instruction, and 65.7% medicine use guide. A higher proportion of rural patients reported having fully utilized blood glucose tests (83.2% vs87.9%, *p* < 0.05) and dietary control instruction (66.6%vs 72.7%, *p* < 0.05) than urban patients (Table 3).

**Table 3:** Patients’ utilization of community-based diabetes management care

Diabetes management service items	Total, n (%)	Urban, n (%)	Rural, n (%)	*P*-value
Fully utilizing blood glucose tests	1853 (85.6)	890 (83.2)	963 (87.9)	0.01^*^
Fully utilizing dietary control instruction	1510 (69.7)	713 (66.6)	797 (72.7)	0.01^*^
Fully utilizing physical excise instruction	1430 (66.0)	697 (65.1)	733 (66.9)	0.21
Fully utilizing medicine use guide	1424 (65.7)	682 (63.7)	742 (67.7)	0.15
Fully utilizing all required diabetes management services in EPHS	1074 (49.6)	520 (48.6)	554 (50.6)	0.36

*Significant at *p* < 0.05; *EPHS* Essential public health services

### Factors associated with the utilization of diabetic management care

Table 4 shows the results of two-level logistic regression models testing the individual- and community-level factors associated with patients’ utilization of diabetes management care in EPHS (partially utilized = 0; fully utilized = 1) among type-2 DM patients. Without including any explanatory variables, 8.0% of the variance in utilization was accounted for at the community level, and there was a significant difference between communities (Model 1). After adding control variables, the community-level variance decreased slightly, but remained significant (Model 2).

**Table 4:** Multilevel logistic regression estimates and variance components of patients’ utilization of community-based management care

Variables	Model 1		Model 2		Model 3		Model 4		Model 5	
	OR,95%CI	P	OR,95%CI	P	OR,95%CI	P	OR,95%CI	P	OR,95%CI	P
**Individual level**										
Area										
Urban (ref)			1		1		1		1	
Rural			1.05 (0.77 ~ 1.43)	0.74	1.15 (0.83 ~ 1.57)	0.40	1.20 (0.89 ~ 1.63)	0.24	1.26 (0.92 ~ 1.72)	0.15
Gender										
Male (ref)			1		1		1		1	
Female			0.91 (0.76 ~ 1.10)	0.35	1.02 (0.84 ~ 1.24)	0.84	0.92 (0.76 ~ 1.10)	0.35	1.02 (0.84 ~ 1.24)	0.85
Age group, years										
< 65 (ref)			1		1		1		1	
≥ 65			0.88 (0.73 ~ 1.05)	0.16	1.06 (0.88 ~ 1.29)	0.53	0.87 (0.73 ~ 1.04)	0.13	1.05 (0.87 ~ 1.28)	0.59
Household income per capita, Yuan										
< 2800			1		1		1		1	
2800~			1.10 (0.86 ~ 1.42)	0.45	0.99 (0.77 ~ 1.29)	0.96	1.11 (0.86 ~ 1.43)	0.42	1.00 (0.77 ~ 1.30)	0.99
6000~			1.06 (0.82 ~ 1.38)	0.65	0.95 (0.73 ~ 1.25)	0.74	1.06 (0.82 ~ 1.38)	0.64	0.96 (0.73 ~ 1.25)	0.74
≥ 12,000			0.93 (0.72 ~ 1.22)	0.62	0.76 (0.58 ~ 1.00)	0.05	0.95 (0.73 ~ 1.24)	0.71	0.78 (0.59 ~ 1.03)	0.08
Duration of diabetes, years										
< 5 (ref)			1		1		1		1	
5 ~ 10			1.16 (0.93 ~ 1.43)	0.18	1.09 (0.88 ~ 1.36)	0.43	1.15 (0.93 ~ 1.42)	0.20	1.09 (0.87 ~ 1.36)	0.45
> 10			1.02 (0.82 ~ 1.27)	0.88	0.86 (0.69 ~ 1.09)	0.21	1.02 (0.82 ~ 1.27)	0.88	0.86 (0.69 ~ 1.09)	0.21
Diabetic knowledge score					1.14 (1.10 ~ 1.17)	0.00			1.14 (1.10 ~ 1.17)	0.00^*^
Diabetic self-efficacy score					1.04 (1.02 ~ 1.06)	0.00			1.04 (1.02 ~ 1.06)	0.00^*^
**Community level**										
Types of PHIs										
Township-level PHIs (ref)							1		1	
Village-level PHIs							1.00 (0.64 ~ 1.55)	0.99	1.10 (0.71 ~ 1.76)	0.63
Number of health workers per 1000 population serviced by PHI							0.02	0.02	1.18 (1.06 ~ 1.30)	0.03*
Healthcare providers knowledge score on diabetes management delivery in PHIs							0.80	0.80	1.02 (0.89 ~ 1.17)	0.76
**Variance components**										
Community level variance	0.287		0.285		0.283		0.257		0.235	
Intra-class correlation	0.080		0.080		0.080		0.072		0.067	

Model 1 is an empty model without any explanatory variables; Model 2 included the control variable at the individual level; Model 3 included the control variables and the important explanatory varied of diabetes knowledge and diabetes self-efficacy at the individual level; Model 4 included the control variables at the individual level and the important variables of primary health institutions at the community level. Model 5 include all the variables at the individual and community levels.

PHIs, primary health institutions; Township-level PHIs included the community health centers in urban areas and the township health centers in rural areas; Village-level PHIs included the community health stations in urban area and the village clinics in urban areas. OR, Odds ratios; CI, confidence interval; *Significant at *p* < 0.05

In Model 3, type-2 DM patients who had better diabetes knowledge (odds ratio, OR = 1.14, 95% confidence interval, *CI*: 1.10 ~ 1.17) and higher self-efficacy in charge of diabetes (OR = 1.04, 95%*CI*: 1.02 ~ 1.06) were more likely to fully utilize community-based diabetes management care. The results were relatively constant even after including community-level variables (Model 5).

In Model 4, the larger number of healthcare providers per 1000 population serviced by the PHIs at community level was associated with higher proportion of the utilization of diabetes management care in EPHS (OR = 1.22, 95%*CI*: 1.03 ~ 1.43). The types of providers and providers’ knowledge regarding diabetes management care delivery were not associated with utilization. After including individual-level factors, the influence of the strength of PHI health workforce remained unchanged (Model 5), though the exact value of OR and CI changed slightly (OR = 1.18, 95%*CI*: 1.06 ~ 1.30). Furthermore, from Models 2 to 5, the variance explained by community-level variables decreased by 19.4%, which implied that these variables had good explanatory power for the variance in patients’ utilization of community-based diabetes management care.

## Discussion

Our findings showed a huge gap between the delivery and utilization of community-based diabetes management care in Shandong province. Half of our participants reported not fully utilizing the required diabetes management services provided by PHIs, regardless of location. Patients’ utilization of community-based diabetes management care was influenced by both patient- and provider-related factors. At individual level, patients’ cognitive and psychological factors, including knowledge of diabetes and self-efficacy in their control of diabetes, were positively associated with the utilization of diabetes management services. At the community level, the number of health workers in PHI per 1000 population was positively associated with utilization.

Globally, community-based diabetes management is known to be an important strategy for the control of diabetes, linked to significant improvement in biophysiological, psychosocial, economic, and adherence outcomes [14, 30]. As the country with the largest diabetic population, China incorporated community-based diabetes management care into EPHS to improve patients’ access to diabetes management care, through organized program management. To encourage adequate service delivery, 50 Yuan (around 7.4 US$) per person was subsided for EPHS in 2017, and a performance-based payment mechanism was also implemented. These incentives had a positive impact on the delivery of community-based diabetes management services [31]. In the current study, all PHIs had reported offering all the diabetes management services required by EPHS, which may point to enhanced availability of services for DM patients. However, our findings also showed that only half the patients, regardless of their location, reported fully utilizing all the required services. Further, the utilization rate established in the current study was significantly lower than those of other integrated health systems, such as the USA’s patient-centered medical home and the integrated diabetes care model in the Netherlands, where up 60% of DM patients obtain regular medical follow-ups from their GPs or diabetes managers [32, 33]. According to China’s National Plan for Non-Communicable Diseases Prevention and Treatment (2017–2025), the utilization rate of diabetes management among DM patients was targeted to be 60% by 2020 and 70% by 2025. Our results imply that more incentives and educational measures should be developed to narrow the gap between the delivery and utilization of community-based diabetes management services in China.

In contrast to previous studies [21, 22], which investigated the factors associated with patients’ utilization of diabetes management care solely from a patient or provider view, the current study explored the factors associated with utilization from both patients’ and providers’ viewpoints, employing multilevel models with patient-related variables at the individual level and provider-related variables at the community level. Our results demonstrated that both provider- and patient-related factors played important roles in patients’ utilization of diabetes management care.

Our analysis of provider-related variables shows that the size of the health workforce in PHIs was positively associated with patients’ utilization of diabetes management care. This is consistent with the results of a study on the impact of health workforce availability on health service use among DM patients in China, which revealed that a higher number of physicians at PHIs had a positive impact on the likelihood of outpatient visits at PHIs [34]. Our results suggest that improvement of the healthcare workforce should be a priority for improving patients’ utilization of diabetes community management services. In China, the government implemented a special education program targeting PHIs, with plans to train 300,000 new physicians over the next 10 years, according to the 2009 health reform plan. However, these plans were thwarted due to increasing medical staff turnover rates [35]. The departure of medical staff from PHIs can mainly be attributed to lower income and fewer career development opportunities [36]. To strengthen the health workforce, appropriate incentive policies, including increased incomes and opportunities for professional development, are needed to attract qualified health workers to work in PHIs. In addition, the integration of PHIs and secondary hospitals and concomitant sharing of human resources may be useful in addressing the shortage of health workers in communities.

In our analysis of patient-related variables, we found that patients’ knowledge on diabetes and their self-efficacy to control diabetes were important predictors of utilization of diabetes management care. This echoes the findings of a study on accessibility among adults with chronic diseases, which showed that psychological accessibility, apart from geographical and economical accessibility, was closely associated to the use of EPHS among individuals with chronic diseases [19]. Knowledge and self-efficacy are two important psychological variables in theory of health behavior [37]. Patients’ diabetes knowledge reflected their cognitive understanding of the risk of diabetic complications and morbidity. Patients’ self-efficacy in controlling diabetes reflects their subjective confidence in maintaining active management in actions to control diabetes. Participants with higher knowledge and self-efficacy may pay closer attention to their diabetes and be more willing to utilize EPHS to comply with diabetes management. However, there were no strong acausal associations between diabetes knowledge and self-efficacy and service utilization due to the cross-sectional design. Some researches had also shown that patients with higher knowledge and self-efficacy were more likely to implement self-management to control blood glucose and obtain health services and self-care instructions from doctors [38]. Therefore, this study hinted that cognitive and psychological factors in patients cannot be ignored in the delivery of community-based diabetes management care. To improve patients’ diabetic knowledge and self-efficacy in control of diabetes, educational and emotional support from health providers, family members and other diabetes patients is needed. Multiple measures aimed at improving communication among providers, patients, family members and peers with diabetes should be implemented to improve patients’ willingness to utilize diabetes management services delivered by PHIs.

### Limitations.

This study has several limitations. First, due to the cross-sectional nature of the study, inferences about causality or temporal ordering of variables cannot be made, such as the relationship between the diabetes knowledge and the utilizations of diabetes management services. Secondly, selection and recall bias might be exist in this study, though numerous quality control measures had been implemented thorough out the study. For the selection bias, the patients who responded to the self-administered questionnaire may be more likely to visits to the PHIs and may have better knowledge of diabetes management. For the recall bias, they may be introduced when patients’ utilization of diabetes management care was measured using self-reported service utilization. Thirdly, self-developed knowledge questionnaires were used in our study to measure patients’ and providers’ knowledge on diabetes. Therefore, our results cannot be feasibly compared with other studies due to non-uniform evaluation criteria. Further, this study did not compare the difference of the provision and utilization of diabetes management services between medical institutions at the same level (town-ship levels or village-levels) due to the smaller sample size, though the difference between the town-ship levels or village-levels PHIs were identified. Finally, this study selected Shandong as the sample setting for the analysis of the contributors to the gap between the delivery and utilization of community-based diabetes management services. While Shandong epitomizes China in terms of population and level of economic development, the issue of representation was not thoroughly considered. A larger sample size should be used in future studies to monitor and evaluate the progress of community-based diabetes management care in China.

## Conclusion

Although the community-based diabetes management services were found be well available in PHIs for Chinese DM patients under the framework of EPHS, our findings show the actual utilization of these services was poor among the patients. Patients’ utilization of diabetes management care depended on both provider- and patient-related factors, including the strength of health workforce in PHIs at community level, and patients’ knowledge of diabetes and self-efficacy to control diabetes at individual level. For developing the community-based care management in China, our study highlights the continuing need for human resource development in PHIs to improve the utilization of services. At the same time, patients’ knowledge and self-efficacy were positively associated with their acceptance and utilization of services. Strengthening health education and promotion of diabetes management services among DM patients may improve their willingness to utilize such care.

## Supplementary information

**Additional file 1.** Text of the Diabetes Knowledge Questionnaire for Patients.

**Additional file 2.** Text of the Diabetes Knowledge Questionnaire for diabetes management services delivery.

## Data Availability

The datasets used and/or analyzed during the current study are available from the corresponding author on reasonable request.

## Abbreviations

CHC: Community health center
CHS: Community health station
DES-SF: Diabetes Empowerment Scale-Short Form
DM: Diabetes mellitus
EPHS: Essential public health services
GDP: Gross domestic product
GP: General practitioner
NCDMS: Non-communicable disease management system
ICC: Intra-class correlation coefficient
PHI: Primary health institution
THC: Township health center
VC: Village clinic
WHO: World Health Organization
