# Supplementary material for: Factors associated with the utilization of community-based diabetes management care: A cross-sectional study in Shandong Province, China
Source: BMC Health Serv Res. 2020 May 11;20:407. doi: 10.1186/s12913-020-05292-5 (PMC7212576; doi:10.1186/s12913-020-05292-5)
Supplement: Supplementary file 1 — Additional file 1. Text of the Diabetes Knowledge Questionnaire for Patients. [file 12913_2020_5292_MOESM1_ESM.docx]

**Appendix 1. Text of the Diabetes Knowledge Questionnaire for Patients**

**DIABETES KNOWLEDGE QUESTIONNAIRE FOR PATIENTS**

**1.** **How many different types of diabetes are there?**

**Please circle ONE answer only**

a. 1 type

b. 2 types

c. ≥3 types*****

d. Unsure

**2.** **What type of diabetes mellitus would you expect to have at the age 4?**

**Please circle ONE answer only**

a. type 1 diabetes*****

b. type 2 diabetes

c. Unsure

**3. People with Over-weight or obesity are more likely to** **develop diabetes than the ones with normal weight.**

**Please circle ONE answer only**

a. Yes*****

b. No

c. Unsure

**4.** **Are people with a family history of diabetes more likely to develop diabetes?**

**Please circle ONE answer only**

a. Yes*****

b. No

c. Unsure

**5. What is the ideal range for blood glucose (sugar) levels a person with diabetes should aim for?**

**Please circle ONE answer only**

a. 2 to 6mmol/L*****

b. 7 to 13mmol/L

c. 4 to 8 mmol/L

d. 4.5 to 15mmol/L

e. Unsure

**6. What were the typical symptoms of diabetes**

**Please circle ONE answer only**

a. excessive urination

b. excessive drinking of water

c. excessive eating

d. unexplained weight loss

d. All of the above*****

e. Unsure

**7. When are patients prone to palpitations, hand tremors, sweating, hunger and other symptoms?**

**Please circle ONE answer only**

a. blood glucose (sugar) levels was much high

b. blood glucose (sugar) levels was much low*****

c. both much high and much low

d. Unsure

**8. Can diabetes cause vision loss or blindness?**

a. Yes*****

b. No

c. Unsure

**9. patients with Diabetes is prone to have itchy skin and Repeated tweezers**

**Please circle ONE option only**

a. Yes*****

b. No

c. Unsure

**10. Which of the following is usually associated with diabetes:**

**Please circle ONE option only**

a. vision problems

b. kidney problems

c. nerve problems

d. foot problems

e. All of the above*****

f. Unsure

**11. Do the patient need to receive treatment when they have no significant diabetic symptoms**

**Please circle ONE option only**

a. Yes*****

b. No

c. Unsure

**12. Why are people with diabetes advised to test their own blood glucose (BG)?**

**Please circle ONE option only**

a. To alert them to changes in BG level patterns

b. To help make decisions in relation to exercise, treating ‘hypos’ (low BG) or sick-day management

c. It can make people more confident in looking after their diabetes

d. All of the above*****

e. Unsure

**13. Do you patients with diabetes need to control your diet to treat their disease?**

**Please circle ONE option only**

a. Yes*****

b. No

c. Unsure

**14. For a person in good control, what effect does exercise have on blood glucose?**

**Please circle ONE option only**

a. Lowers it*****

b. Raises it

c. Has no effect

d. Unsure

**15 Can diabetes be cured?**

**Please circle ONE option only**

a. Yes

b. No*****

c. Unsure

**16. Can diabetes medication be stopped If blood glucose levels are normal for two months?**

**Please circle ONE option only**

a. Yes

b. No*****

c. Unsure

*** Correct answer**
