# Supplementary material for: Factors associated with the utilization of community-based diabetes management care: A cross-sectional study in Shandong Province, China
Source: BMC Health Serv Res. 2020 May 11;20:407. doi: 10.1186/s12913-020-05292-5 (PMC7212576; doi:10.1186/s12913-020-05292-5)
Supplement: Supplementary file 2 — Additional file 2. Text of the Diabetes Knowledge Questionnaire for diabetes management services delivery. [file 12913_2020_5292_MOESM2_ESM.docx]

**Appendix 2. Text of the Diabetes Knowledge Questionnaire for diabetes management services delivery**

**DIABETES KNOWLEDGE QUESTIONNAIRE FOR PROVIDERS**

**1. WHO diagnostic criteria for diabetes were:**

**Please circle ONE answer only**

a. FPG≥7.0mmol/L

b.75g OGTT with FPG≥7.0mmol/L and/or 2-hour plasma glucose≥11.1 mmol/L

c.HbA_1C_≥6.5%

d. Random plasma glucose≥11.1 mmol/L

e. All of the above*

f. Unsure

**2. The risk factors for diabetes included:**

**Please circle ONE answer only**

a. Smoking

b. Obesity

c. Sedentary lifestyle

d. Family history of diabetes

e. All of the above*

f. Unsure

**3. diabetes increases the risk of:**

**Please circle ONE answer only**

a. Kidney damage

b. Blindness

c. Heart disease

d. Foot ulcers

e. All of the above*

f. Unsure

**4. Diabetes is a condition that: Please circle ONE answer only**

**Please circle ONE answer only**

a. Can be cured by adopting a healthy lifestyle

b. Can be cured with tablets and/or insulin

c. Is currently not curable*

d. Is always life threatening when first diagnosed

e. Unsure

**5. The first treatment that should be considered when a patient is diagnosed with type 2 diabetes is?**

**Please circle ONE answer only**

a. Diet control &physical exercise*

b. insulin use

c. Oral hypoglycemic agent use

d. Diet control & Oral hypoglycemic agent use

e. Unsure

**6. What foods are not suitable for diabetics?**

**Please circle ONE answer only**

a. Jam*

b. cereals

c. vegetables

d. low-fat diet

e. Unsure

7. which one is the food with highest glycemic index (GI)?

**Please circle ONE answer only**

a. watermelon*

b. potato

c. tomato

d. apple

e. Unsure

**8. The daily salt intake of diabetic patients should not exceed:**

**Please circle ONE answer only**

a. 5g

b. 6g*

c. 7g

d. 8g

e. Unsure

**9. How often should people with diabetes exercise or be physically active?**

**Please circle ONE answer only**

a. Most days of the week for at least 30 minutes*

b. Once a week for at least 30 minutes

c. Once a month for one hour

d. At least every fortnight for two hours

e. Unsure

**10.** **Diabetes patients should not excise when they…**

**Please circle ONE answer only**

a. FPG≥16.7mmol/L

b. had severe DKA

c. Have severe kidney disease

d. Have severe cardiovascular and cerebrovascular diseases

e. All of the above*

f. Unsure

**11. Which of the following sports is suitable for middle-aged and elderly diabetic patients?**

**Please circle ONE answer only**

a. Weightlifting

b. Racing

c. Chinese Tai Chi*

d. Swimming

e. Unsure

**12. which the tablet was not hypoglycemic drugs**

**Please circle ONE answer only**

a. Metformin

b. Glibenclamide

c. Acarbose

d. Nifedipine*

e. Unsure

**13. which tablet is easy to cause hypoglycemia when used alone?**

**Please circle ONE answer only**

a. Repaglinide

b. Acarbose

c. Sitagliptin

d. Glibenclamide*

e. Unsure

**14. What kind of vitamin should be added to diabetic patients who take metformin for a long time?**

**Please circle ONE answer only**

a. Vitamin A

b. Vitamin B_12_*

c. Vitamin C

d. vitamin D

e. Unsure

**15. What is the ideal range for blood glucose (sugar) levels a person with diabetes should aim for?**

**Please circle ONE answer only**

a. 2 to 6mmol/L*

b. 7 to 13mmol/L

c. 4 to 8 mmol/L

d. 4.5 to 15mmol/L

e. Unsure

**16. A1C is a measure of average blood glucose level for the past:**

**Please circle ONE answer only**

a. day

b. week

c. 6-12 weeks*

d. 6 months

e. Unsure

**17. Which blood glucose monitoring method can be used to reflect the basic secretion function of human insulin?**

**Please circle ONE answer only**

a. A1C

b. FPG*

c. Blood sugar between 1 and 3 a.m

d. Bedtime blood glucose

e. Unsure

**18. What effect will an infection most likely have on blood glucose?**

**Please circle ONE answer only**

a. Lowers it

b. Raises it*

c. Has no effect

d. Unsure

**19. Which of the following symptoms is likely to occur in patients with hypoglycemia?**

**Please circle ONE answer only**

a. More urine;

b. Extreme thirst

c. Palpitation, dizziness, cold sweat*

d. Skin itching

e. Unsure

**20. If a patient with diabetes has a hypo (low blood glucose level) reaction, s/he should:**

**Please circle ONE answer only**

a. Immediately take some insulin or diabetes tablets

b. Rest and wait until s/he feels better

c. Immediately have some sugary food or drink (e.g. jelly beans, soft drink) *

d. Drink some diet soft drink

e. Unsure

**21. What kind of shoes are suitable for diabetics?**

**Please circle ONE answer only**

a. leather shoes

b. shoes with high heels

c. loose cloth shoes*

d. as bare as possible

e. Unsure

**22. Which is the best way that the diabetes patients take care of their feet?**

**Please circle ONE answer only**

a. Look at and wash them each day*

b. Massage them with alcohol each day

c. Soak them for 1 hour each day

d. Buy shoes a size larger than usual

e. Unsure

*** Correct answer**
